# DNA methylation mediated *RSPO2* to promote follicular development in mammals

**DOI:** 10.1038/s41419-021-03941-z

**Published:** 2021-06-26

**Authors:** Xiaofeng Zhou, Yingting He, Nian Li, Guofeng Bai, Xiangchun Pan, Zhe Zhang, Hao Zhang, Jiaqi Li, Xiaolong Yuan

**Affiliations:** 1 Guangdong Laboratory of Lingnan Modern Agriculture, National Engineering Research Center for Breeding Swine Industry, Guangdong Provincial Key Lab of Agro-Animal Genomics and Molecular Breeding, College of Animal Science, South China Agricultural University, Guangzhou, Guangdong China; 2 Guangdong Provincial Key Laboratory of Laboratory Animals, Guangdong Laboratory Animals Monitoring Institute, Guangzhou, China

**Keywords:** Apoptosis, Transcription

## Abstract

In female mammals, the proliferation, apoptosis, and estradiol-17β (E2) secretion of granulosa cells (GCs) have come to decide the fate of follicles. DNA methylation and *RSPO2* gene of Wnt signaling pathway have been reported to involve in the survival of GCs and follicular development. However, the molecular mechanisms for how DNA methylation regulates the expression of *RSPO2* and participates in the follicular development are not clear. In this study, we found that the mRNA and protein levels of *RSPO2* significantly increased during follicular development, but the DNA methylation level of *RSPO2* promoter decreased gradually. Inhibition of DNA methylation or *DNMT1* knockdown could decrease the methylation level of CpG island (CGI) in *RSPO2* promoter and upregulate the expression level of *RSPO2* in porcine GCs. The hypomethylation of −758/−749 and −563/−553 regions in *RSPO2* promoter facilitated the occupancy of transcription factor *E2F1* and promoted the transcriptional activity of *RSPO2*. Moreover, *RSPO2* promoted the proliferation of GCs with increasing the expression level of *PCNA*, *CDK1*, and *CCND1* and promoted the E2 secretion of GCs with increasing the expression level of *CYP19A1* and *HSD17B1* and inhibited the apoptosis of GCs with decreasing the expression level of *Caspase3*, cleaved *Caspase3*, cleaved *Caspase8*, cleaved *Caspase9*, cleaved *PARP*, and *BAX*. In addition, *RSPO2* knockdown promoted the apoptosis of GCs, blocked the development of follicles, and delayed the onset of puberty with decreasing the expression level of Wnt signaling pathway-related genes (*LGR4* and *CTNNB1*) in vivo. Taken together, the hypomethylation of −758/−749 and −563/−553 regions in *RSPO2* promoter facilitated the occupancy of *E2F1* and enhanced the transcription of *RSPO2*, which further promoted the proliferation and E2 secretion of GCs, inhibited the apoptosis of GCs, and ultimately ameliorated the development of follicles through Wnt signaling pathway. This study will provide useful information for further exploration on DNA-methylation-mediated *RSPO2* pathway during follicular development.

## Introduction

In female mammals, the normal follicular development and ovarian maturation are essential for the initiation of puberty and acquisition of the capacity to fertilization and reproduction [1, 2]. In mice, knocking out the *KISS1* gene, which is well known for the gate regulator of pubertal timing, resulted in the absence of mature follicles and failure of pubertal transition [3]. Previous studies have shown that the proliferation and apoptosis of granulosa cells (GCs) as well as the synthesis of estradiol-17β (E2) are required for the follicular development [4–6]. The excessive apoptosis and death of GCs is quite possible to directly or indirectly block follicular development [7, 8]. For example, the disruption of estrogen receptor beta expression in mutant mice reduces the E2 production of GCs [9, 10], increases the apoptosis of GCs, and results in the failure of follicular maturation [11] and ovulatory dysfunction [12, 13]. In premature ovarian failure mice, upregulation of anti-Müllerian hormone expression in GCs inhibits the apoptosis of GCs and promotes the follicular development [14]. These observations suggest that the proliferation, apoptosis, and secretion of E2 in GCs have come to decide the fate of follicles in mammals. However, the mechanisms by which the GCs regulate the follicular development and pubertal initiation have not been fully understood.

Accumulating studies demonstrate the crucial roles of DNA methylation in the follicular development and the onset of puberty [15, 16]. DNA methylation is adding the methyl on the cytosine residues to regulate the transcription of genes mainly by DNA methyltransferases 1 (*DNMT1*) [17]. Previous studies have shown that the disrupted DNA methylation contributes to the arrest of follicular development and delay of puberty in rats [18, 19]. *DNMT1*-mediated MEG3 hypermethylation has been related to puberty by inhibiting the P53 signaling pathway [20, 21]. In polycystic ovary syndrome (PCOS) patients, *DNMT1*-dependent *CDKN1A* promoter hypomethylation inhibits the proliferation and growth of GCs [22]. In bovine luteal cells, the hypermethylation of the *CYP19A1* gene promoter inhibits its expression to suppress the production of E2 [23, 24]. In rats, the hypomethylation of *Caspase3* promoter promotes its expression and leads to the apoptosis of GCs [25]. LINE1 CpG-DNA hypomethylation in women GCs is strongly associated with PCOS [26]. In cumulus GCs of women with PCOS, the hypermethylation of *TNF* inhibits its expression to hinder cumulus oocyte complex expansion and compromise ovulation [27]. In summary, the change of DNA methylation in GCs may participate in follicular development and pubertal initiation by regulating the expression of key genes.

The *RSPO2* gene is a member of the R-Spondin family, which activates and strongly potentiates the Wnt signaling pathway through their homologous *LGR4*, *LGR5*, and *LGR6* receptors [28–30]. In mammals, *RSPO* family plays an important role in ovarian cell proliferation, apoptosis, and follicular development [31–33]. For example, interfering with the transcription of *RSPO1* inhibits the cellular proliferation and promotes cellular apoptosis in human ovarian cancer cells [31]. *RSPO2* promotes the transformation of primary follicles into secondary follicles in mouse ovaries [32]. Knockout *RSPO2* inhibits the proliferation and differentiation of mouse ovarian GCs and impedes the growth of follicles [33]. In this study, we found that two CpG islands (CGIs) existed in the promoter of *RSPO2*. Therefore, we hypothesized that DNA methylation might be involved in the proliferation, apoptosis, and E2 secretion of porcine GCs by mediating the expression of *RSPO2*, thus affecting the follicular development and onset of puberty in mammals.

## Results

### DNA hypomethylation in CGI2 promoted the expression level of *RSPO2* during follicular development

**Fig. 1:**
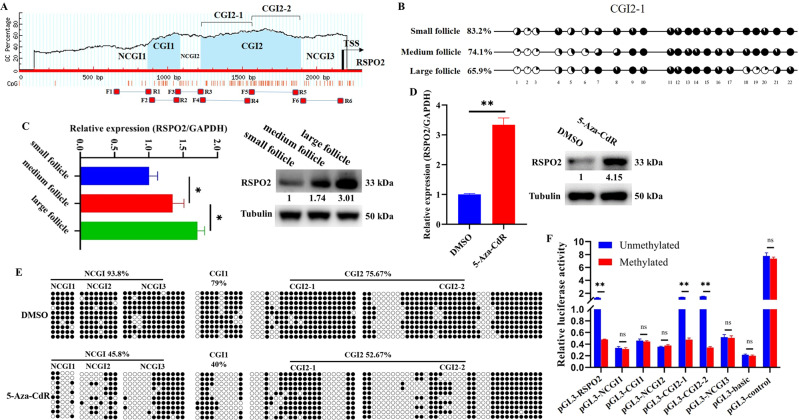
DNA hypomethylation in the CGI2-1 region of *RSPO2* promoter might promote the expression level of *RSPO2* during follicular development. **A** Schematic distribution of the CpG islands and bisulfite sequencing primers in the promoter of *RSPO2*. Blue region is the CpG island; red and horizontal bars show the positions of bisulfite primers. **B** Bisulfite sequencing was conducted to determine the methylation status of CGI2-1 at the *RSPO2* promoter in small (1–3 mm), medium (3–5 mm), and large (5–7 mm) follicles. **C** The mRNA and protein expression level of *RSPO2* in small, medium, and large follicles. **D** The mRNA and protein expression level of *RSPO2* in porcine GCs treated with 5-Aza-CdR. The numbers below bands were folds of band intensities relative to control. The band intensities were measured by ImageJ and normalized to Tubulin. **E** BSP was used to detect the NCGI and CGI methylation status of *RSPO2* in porcine GCs treated with 5-Aza-CdR. **F** The luciferase reporter plasmids carrying different regions of *RSPO2* were methylated in vitro and transfected into GCs. Relative luciferase activity in GCs were determined. *P < 0.05, **P < 0.01. CGI CpG island, NCGI non-CpG island.

In order to explore whether DNA methylation was involved in the expression of *RSPO2* during follicular development, the methylation and expression levels of *RSPO2* were detected. There were two CGIs in the promoter of *RSPO2* (CGI1, 217 base pair (bp), −1437/−1220 bp and CGI2, 669 bp, −1078/−409 bp, transcription start site = +1; Fig. 1A). During the development of porcine follicles, the DNA methylation levels of CGI2-1 decreased gradually across small follicles (1–3 mm), medium follicles (3–5 mm), and large follicles (5–7 mm) (Fig. 1B), but the mRNA and protein expression levels of *RSPO2* significantly increased (Fig. 1C). To further confirm the regulation of DNA methylation on the expression of *RSPO2*, the expression and methylation levels of *RSPO2* in GCs were examined after treating with DNA methylation inhibitor (5-Aza-CDR). We found that inhibition of DNA methylation significantly upregulated the mRNA and protein expression levels of *RSPO2* in GCs (Fig. 1D) and reduced the methylation levels of non-CpG islands (NCGIs) and CGIs at *RSPO2* promoter (Fig. 1E). Furthermore, to identify the core regulatory region of DNA-methylation-mediated *RSPO2* expression, we segmented and methylated the NCGIs (pGL3-NCGI1: −1540/−1357 bp, pGL3-NCGI2: −1187/−1003 bp, and pGL3-NCGI3: −327/−76 bp) and CGIs (pGL3-CGI1: −1345/−1165 bp, pGL3-CGI2-1: −1014/−721 bp, and pGL3-CGI2–2: −709/−362 bp) of *RSPO2* promoter. Dual-luciferase activity analysis showed that the methylated pGL3-CGI2-1 and pGL3-CGI2–2 significantly reduced the transcriptional activity of *RSPO2*, compared with the unmethylated pGL3-CGI2-1 and pGL3-CGI2–2. But the methylated pGL3-NCGI1, pGL3-CGI1, pGL3-NCGI2, and pGL3-NCGI3 did not exhibit marked effect on the transcriptional activity of *RSPO2* (Fig. 1F). These results indicated that the hypomethylation in CGI2 promoted the expression of *RSPO2* during follicular development.

### *RSPO2* promoted the proliferation of porcine ovarian GCs

**Fig. 2:**
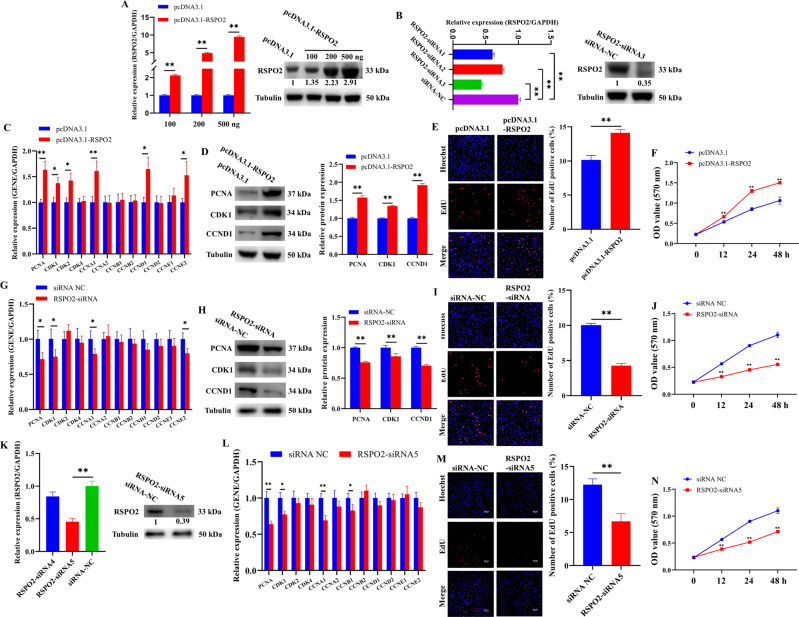
*RSPO2* promoted the proliferation of porcine ovarian GCs. **A**, **B** The mRNA and protein expression levels of *RSPO2* after transfection with pcDNA3.1-*RSPO2* (**A**) or *RSPO2*-siRNA (**B**) in porcine GCs. The numbers below bands were folds of band intensities relative to control. The band intensities were measured by ImageJ and normalized to Tubulin. **C**, **D** The mRNA (**C**) and protein (**D**) expression levels of *PCNA*, *CDK1*, and *CCND1* in porcine GCs with *RSPO2* overexpression. **E**, **F** The proliferation rates of GCs with *RSPO2* overexpression were assessed by EdU (**E**) and MTT assay (**F**). **G**, **H** The mRNA (**G**) and protein (**H**) expression levels of *PCNA*, *CDK1*, and *CCND1* in porcine GCs with *RSPO2* knockdown. **I**, **J** The proliferation rates of GCs with *RSPO2* knockdown were assessed by EdU (**I**) and MTT assay (**J**). **K** The mRNA and protein expression levels of *RSPO2* after transfection with another two *RSPO2*-siRNAs (*RSPO2*-siRNA4 and 5) in porcine GCs. **L** The mRNA expression levels of cell cycle-related genes in porcine GCs transfected with *RSPO2*-siRNA5. **M**, **N** The proliferation rates of GCs transfected with *RSPO2*-siRNA5 was assessed by EdU (**M**) and MTT assay (**N**). *P < 0.05, **P < 0.01.

**Fig. 3:**
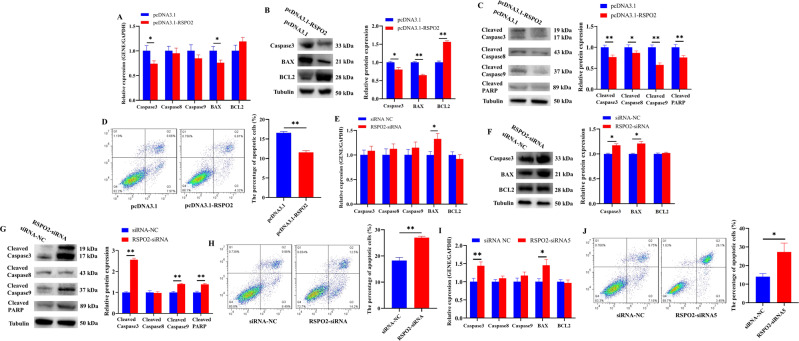
*RSPO2* inhibited the apoptosis of porcine ovarian GCs. **A**–**C** The mRNA (**A**) and protein (**B**) expression levels of *Caspase3*, *Caspase8*, *Caspase9*, *BAX*, and *BCL2*, as well as the cleaved protein (**C**) expression levels of *Caspase3*, *Caspase8*, *Caspase9*, and *PARP* in porcine GCs with *RSPO2* overexpression. **D** The apoptosis rates of GCs with *RSPO2* overexpression was assessed by flow cytometry. **E**–**G** The mRNA (**E**) and protein (**F**) expression levels of *Caspase3*, *Caspase8*, *Caspase9*, *BAX*, and *BCL2*, as well as the cleaved protein (**G**) expression levels of *Caspase3*, *Caspase8*, *Caspase9*, and *PARP* in porcine GCs with *RSPO2* knockdown. **H** The apoptosis rates of GCs with *RSPO2* knockdown was assessed by flow cytometry. **I** The mRNA expression levels of *Caspase3*, *Caspase8*, *Caspase9*, *BAX*, and *BCL2* in porcine GCs transfected with *RSPO2*-siRNA5. **J** The apoptosis rates of GCs transfected with *RSPO2*-siRNA5 was assessed by flow cytometry. *P < 0.05, **P < 0.01.

**Fig. 4:**
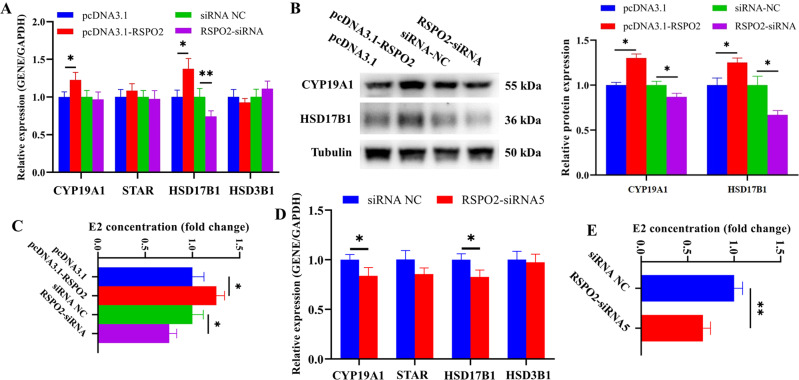
*RSPO2* promoted the secretion of E2 in porcine ovarian GCs. **A, B** The mRNA (**A**) and protein (**B**) expression levels of *CYP19A1*, *STAR*, *HSD17B1*, and *HSD3B1* in porcine GCs with *RSPO2* overexpression and inhibition. **C** The concentration of E2 in porcine ovarian GCs with *RSPO2* overexpression and inhibition were assessed by ELISA. **D** The mRNA expression levels of *CYP19A1*, *STAR*, *HSD17B1*, and *HSD3B1* in porcine GCs transfected with *RSPO2*-siRNA5. **E** The concentration of E2 in porcine ovarian GCs transfected with *RSPO2*-siRNA5 was assessed by ELISA. *P < 0.05, **P < 0.01.

To determine the effect of *RSPO2* on the proliferation of GCs, *RSPO2* overexpression vector (pcDNA3.1-*RSPO2*) or *RSPO2* small interfering RNA (*RSPO2*-siRNA) were transfected into GCs, and the proliferation ability and expression of genes related to proliferation pathways were detected. We initially confirmed the efficiency of *RSPO2* overexpression and knockdown through quantitative reverse transcription PCR (qRT-PCR) and western blotting (WB). The mRNA and protein expression levels of *RSPO2* in GCs increased significantly after transfecting with pcDNA3.1-*RSPO2* (Fig. 2A), and the overexpression effect of *RSPO2* increased with the increase of vector concentration and 500 ng of pcDNA3.1-*RSPO2* was selected for subsequent experiments as it exhibited the strongest overexpression efficiency. Three *RSPO2*-siRNAs (*RSPO2*-siRNA1, *RSPO2*-siRNA2, and *RSPO2*-siRNA3) were synthesized and transfected into GCs for *RSPO2* knockdown. *RSPO2*-siRNA3 significantly reduced the mRNA and protein expression levels of *RSPO2* (Fig. 2B) and was selected for subsequent experiments as it exhibited the strongest knockdown efficiency. The qRT-PCR (Fig. 2C) and WB (Fig. 2D) showed that *RSPO2* overexpression significantly promoted the mRNA expression level of cell cycle-related genes (*PCNA*, *CDK1*, *CDK2*, *CCNA1*, *CCND1*, and *CCNE2*) as well as the protein expression of *PCNA*, *CDK1*, and *CCND1*. Moreover, 5-ethynyl-2′-deoxyuridine (EdU) staining (Fig. 2E) and 3-[4,5-dimethylthiazol-2-yl]-2,5 diphenyl tetrazolium bromide (MTT) assay (Fig. 2F) showed that *RSPO2* overexpression significantly increased the proliferation ability of GCs. Meanwhile, *RSPO2* silencing significantly inhibited the mRNA expression level of *PCNA*, *CDK1*, *CCNA1*, and *CCNE2* as well as the protein expression of *PCNA*, *CDK1*, and *CCND1* (Fig. 2G, H) and significantly inhibited the proliferation of GCs (Fig. 2I, J). Furthermore, we synthesized and transfected GCs with *RSPO2*-siRNA4 and *RSPO2*-siRNA5 to avoid off-target effect-related phenotype. Based on the knockdown efficiency, *RSPO2*-siRNA5 was selected and significantly reduced the mRNA and protein expression levels of *RSPO2* (Fig. 2K) and inhibited the mRNA expression of *PCNA*, *CDK1*, *CCNA1*, and *CCNB1* (Fig. 2L). EdU staining (Fig. 2M) and MTT assay (Fig. 2N) demonstrated that *RSPO2* knockdown by *RSPO2*-siRNA5 significantly depressed the proliferation of GCs. Taken together, these results suggested that *RSPO2* promoted the proliferation of porcine ovarian GCs.

### *RSPO2* inhibited the apoptosis of porcine ovarian GCs

To determine the effect of *RSPO2* on the apoptosis of GCs, the apoptosis rate and expression of genes related to apoptosis pathways were detected after overexpression and knockdown of *RSPO2*. *RSPO2* overexpression significantly inhibited the mRNA and protein expression level of pro-apoptosis-related genes (*Caspase3* and *BAX*; Fig. 3A, B) and significantly promoted the protein expression levels of anti-apoptosis-related gene (*BCL2*; Fig. 3B). To further confirm the effects of *RSPO2* on apoptosis, the cleaved protein expression of *Caspase3*, *Caspase8*, *Caspase9*, and *PARP* in GCs were detected. Results showed that *RSPO2* overexpression significantly inhibited the protein expression of cleaved *Caspase3*, cleaved *Caspase8*, cleaved *Caspase9*, and cleaved *PARP* (Fig. 3C) and the apoptosis of GCs (Fig. 3D). Moreover, *RSPO2* silencing significantly promoted the mRNA expression level of *BAX* (Fig. 3E) as well as the protein expression level of *Caspase3*, *BAX*, cleaved *Caspase3*, cleaved *Caspase9*, and cleaved *PARP* (Fig. 3F, G) and significantly promoted the apoptosis of GCs (Fig. 3H). Similarly, *RSPO2*-siRNA5 also significantly promoted the mRNA expression of *Caspase3* and *BAX* (Fig. 3I) as well as the apoptosis of GCs (Fig. 3J). Collectively, these results suggested that *RSPO2* inhibited the apoptosis of porcine ovarian GCs.

### *RSPO2* promoted the secretion of E2 in porcine ovarian GCs

To determine the effect of *RSPO2* on the E2 secretion of GCs, the expression of genes related to estrogen signaling pathway were detected after overexpression and knockdown of *RSPO2*. qRT-PCR (Fig. 4A) and WB (Fig. 4B) results indicated that *RSPO2* overexpression significantly promoted the mRNA and protein levels expression of *CYP19A1* and *HSD17B1*, while *RSPO2* silencing significantly inhibited the mRNA and protein expression levels of *HSD17B1* as well as the protein expression level of *CYP19A1*. Moreover, *RSPO2* overexpression significantly promoted the secretion of E2 in GCs, while *RSPO2* knockdown displayed the opposite effects (Fig. 4C). Similarly, *RSPO2*-siRNA5 also significantly inhibited the mRNA expression of *CYP19A1* and *HSD17B1* (Fig. 4D) and the secretion of E2 in GCs. (Fig. 4E). In summary, *RSPO2* promoted the secretion of E2 in porcine ovarian GCs.

### Inhibition of *RSPO2* blocked the development of porcine follicles

**Fig. 5:**
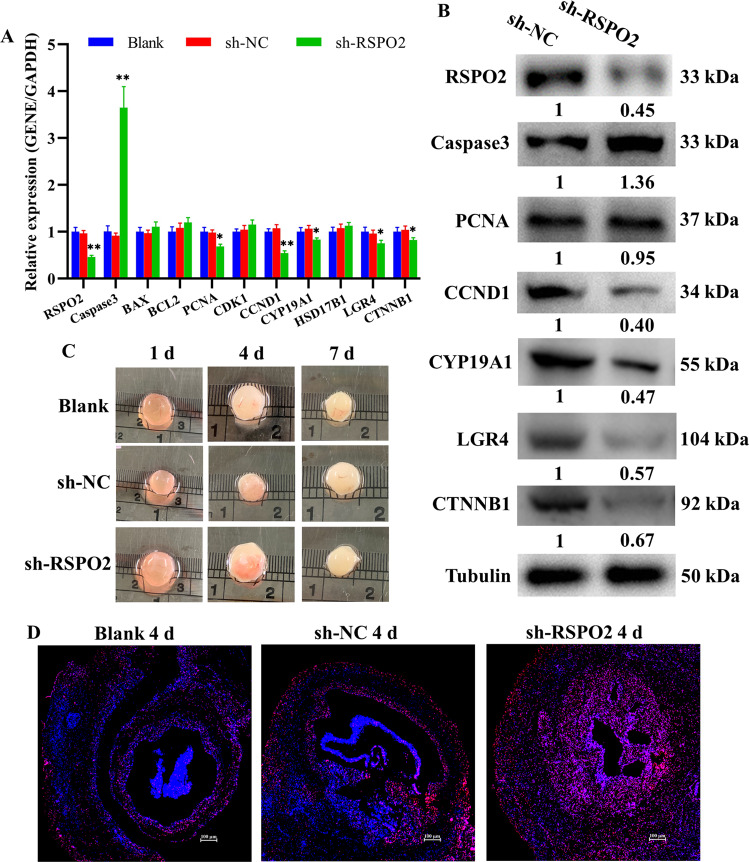
*RSPO2* knockdown blocked the development of porcine follicles. **A, B** The mRNA (**A**) and protein (**B**) expression levels of *RSPO2*, *Caspase3*, *PCNA*, *CCND1*, *CYP19A1*, *LGR4*, and *CTNNB1* in porcine follicle with *RSPO2* inhibition. The numbers below bands were folds of band intensities relative to control. The band intensities were measured by ImageJ and normalized to Tubulin. **C** The photos of porcine follicles cultured in vitro after transfected with sh-*RSPO2* on first, fourth, and seventh day. **D** The apoptosis of follicular GCs was detected by TUNEL after transfected with sh-*RSPO2* at the fourth day. *P < 0.05, **P < 0.01.

To further investigate the biological function of *RSPO2* in the development of follicles, the lentiviral vector knockdown of *RSPO2* (sh-*RSPO2*) and negative control (sh-NC) were built and transfected into porcine follicles cultured in vitro. The mRNA and protein expression levels of *RSPO2* in sh-*RSPO2* group were significantly lower than those in the sh-NC group (Fig. 5A, B), indicating that sh-*RSPO2* has been successfully transfected into follicles. Moreover, *RSPO2* knockdown decreased the mRNA and protein expression levels of *PCNA*, *CCND1*, *CYP19A1*, *LGR4*, and *CTNNB1*, while increased the mRNA and protein expression levels of *Caspase3* in porcine follicles (Fig. 5A, B). Besides, *RSPO2* knockdown promoted the loss of follicular blood vessels and the opacity of follicular fluid (Fig. 5C), and terminal deoxynucleotidyl transferase-mediated dUTP-fluorescein nick end labeling (TUNEL) assay showed that the apoptosis of GCs was markedly increased (Fig. 5D). These results indicated that inhibition of *RSPO2* might block the development of porcine follicles by promoting the apoptosis of GCs.

### Silencing *RSPO2* blocked follicular development and onset of puberty in mice

**Fig. 6:**
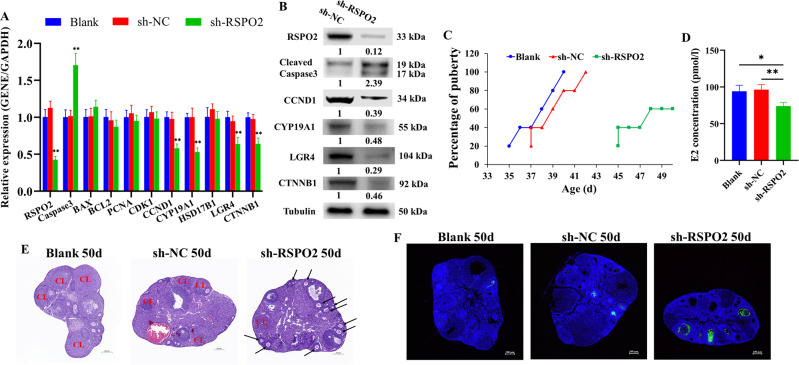
Silencing *RSPO2* blocked the follicular development and onset of puberty in mice. **A**, **B** The mRNA (**A**) and protein (**B**) expression levels of *RSPO2*, *Caspase3*, cleaved *Caspase3*, *CCND1*, *CYP19A1*, *LGR4*, and *CTNNB1* in the ovaries of mice with *RSPO2* inhibition. The numbers below bands were folds of band intensities relative to control. The band intensities were measured by ImageJ and normalized to Tubulin. **C** The percentage of mice at vaginal opening in the blank, sh-NC, and sh-*RSPO2* groups (*n* = 5). **D** The E2 concentration in serum from blank, sh-NC and sh-*RSPO2*-injected mice collected at age 50 days was detected by ELISA. **E** Example of the ovaries from blank, sh-NC, and sh-*RSPO2*-injected mice collected at age 50 days. CL indicates corpora lutea and arrows point to example of antral follicles. **F** The apoptosis of follicular GCs from blank, sh-NC, and sh-*RSPO2*-injected mice collected at age 50 days was detected by TUNEL. *P < 0.05, **P < 0.01.

To further verify the role of *RSPO2* on follicular development and the onset of puberty, the sh-*RSPO2* lentiviral vector was injected into mice via intraperitoneal injection. Then the mice were slaughtered, and we found that the mRNA and protein expression levels of *RSPO2* in the ovaries of mice transfected with sh-*RSPO2* were significantly lower than those mice transfected with sh-NC (Fig. 6A, B), indicating that sh-*RSPO2* had been successfully transfected into mice. Meanwhile, *RSPO2* knockdown decreased the mRNA and protein expression levels of *CCND1*, *CYP19A1*, *LGR4*, and *CTNNB1*, while increased the mRNA expression level of *Caspase3* as well as protein expression level of cleaved *Caspase3* in the mouse ovaries (Fig. 6A, B). Next, the age of pubertal initiation and serum E2 level of mice transfected with sh-*RSPO2* were analyzed, the development status of ovarian follicles was detected by hematoxylin and eosin staining, and the apoptosis of GCs in follicles was examined by TUNEL. The mice injected with sh-*RSPO2* had delayed vaginal opening (Fig. 6C; mean age at vaginal opening: blank, 38 ± 2.12 days; sh-NC, 38.8 ± 1.48 days; sh-*RSPO2*, 46.8 ± 2.68 days; *P* < 0.01). *RSPO2* knockdown significantly decreased the serum E2 concentration in mice (Fig. 6D). Examination of the ovaries at age 50 days showed that sh-*RSPO2*-treated mice had numerous antral follicles but few corpora lutea, indicating that they had not ovulated and consequently that pubertal initiation failed (Fig. 6E). In contrast, the ovaries of sh-NC-treated and untreated mice exhibited many corpora lutea but few antral follicles, indicating that they ovulated and the pubertal initiation had occurred (Fig. 6E). Moreover, *RSPO2* knockdown increased the apoptosis of ovarian GCs in mice (Fig. 6F). These results indicated that silencing *RSPO2* might inhibit the development of follicles and eventually delay the onset of puberty by promoting the apoptosis of GCs and inhibiting the secretion of E2 in mice.

### Hypomethylation of CGI2 recruited *E2F1* to promote the transcription of *RSPO2*

**Fig. 7:**
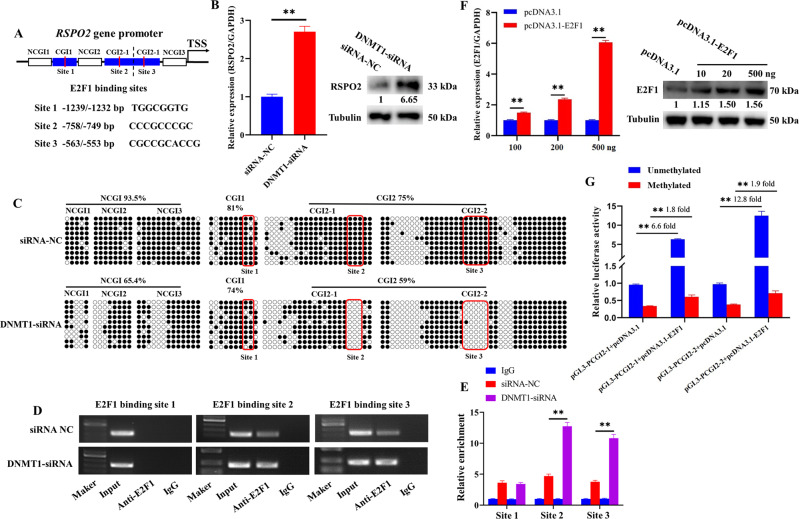
Hypomethylation of CGI2 recruited *E2F1* to promote the transcription of *RSPO2*. **A** Predicted *E2F1*-binding sites in the CGI1 and CGI2 region of *RSPO2* promoter. **B** The mRNA and protein expression level of *RSPO2* in porcine GCs treated with DNMT1-siRNA. The numbers below bands were folds of band intensities relative to control. The band intensities were measured by ImageJ and normalized to Tubulin. **C** BSP was used to detect the NCGI and CGI methylation status of *RSPO2* in porcine GCs treated with DNMT1-siRNA. The red box showed the three potential binding sites region of *E2F1* in the *RSPO2* promoter CGI. **D**, **E** The enrichment of *E2F1* on the CGI region after *DNMT1* knockdown was detected by ChIP (**D**) and ChIP-qPCR (**E**). **F** The mRNA and protein expression levels of *E2F1* after transfection with pcDNA3.1 or pcDNA3.1-*E2F1* in porcine GCs. **G** The luciferase reporter plasmids carrying CGI2 region of *RSPO2* were methylated in vitro and co-transfected with pcDNA3.1-*E2F1* into GCs. Relative luciferase activity in GCs was determined. **P < 0.01.

To explore the mechanism of DNA methylation that regulated *RSPO2* expression, PROMO website was used to predict the potential transcription factor-binding sites of the CGI region. There were one potential binding site of *E2F1* on the CGI1 region (Site1, −1239/−1232 bp, Fig. 7A) and two potential binding sites of *E2F1* on the CGI2 region (Site2, −758/−749 bp and Site3, −563/−553 bp, Fig. 7A). Then the expression of *RSPO2* in GCs treated with *DNMT1*-siRNA was detected, and *DNMT1* knockdown significantly increased the mRNA and protein levels of *RSPO2* (Fig. 7B). Bisulfite sequencing PCR (BSP) was used to measure the methylation level changes of NCGI1, NCGI2, NCGI3, CGI1, CGI2-1, and CGI2-2 after *DNMT1*. As shown in Fig. 7C, the methylation levels of Site2 and Site3 were obviously reduced, but there was no difference at Site1 (Fig. 7C). Chromatin immunoprecipitation (ChIP) and ChIP-qPCR were further performed to detect whether the occupancy of *E2F1* to CGI was regulated by DNA methylation. Results showed that the enrichment and occupancy of *E2F1* at Site2 and Site3 were significantly increased after *DNMT1* knockdown, but there was no difference at Site1 (Fig. 7D, E). These observations suggested that DNA methylation might regulate RSPO2 expression by mediating the occupancy of E2F1 to CGI2 at Site2 and Site3.

Moreover, the overexpression vector of *E2F1* (pcDNA3.1-*E2F1*) were constructed, and the effect of *E2F1* on the transcriptional activity of *RSPO2* with methylated or unmethylated CGI2 were detected by dual-luciferase activity analysis. *E2F1* overexpression significantly increased the mRNA and protein levels of *E2F1* in GCs (Fig. 7F). Furthermore, *E2F1* overexpression significantly increased the transcription activity of the unmethylated pGL3-CGI2-1 by 6.6 times, while only 1.8 times in the methylated pGL3-CGI2-1. Similarly, *E2F1* overexpression significantly increased the transcription activity of the unmethylated pGL3-CGI2–2 by 12.8 times, while only 1.9 times in the methylated pGL3-CGI2–2 (Fig. 7G). These results implied that the hypomethylation of CGI2 recruited *E2F1* to promote the transcription of *RSPO2*.

## Discussion

**Fig. 8:**
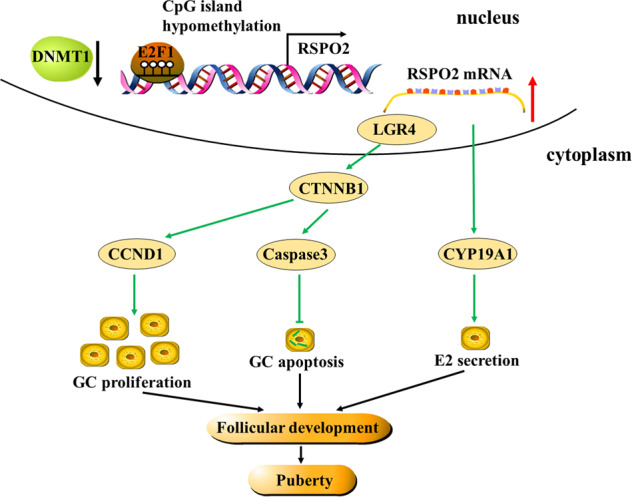
The mechanistic scheme of DNA-methylation-mediated RSPO2 regulating GCs proliferation, apoptosis and E2 secretion. The hypomethylation of CGI2 in RSPO2 promoter facilitated the occupancy of E2F1 and enhanced the transcription of RSPO2, which further promoted the proliferation and E2 secretion of GCs, inhibited the apoptosis of GCs and ultimately ameliorated the development of follicles and pubertal initiation through Wnt signaling pathway.

Accumulating studies have shown that the survival of GCs determines the fate of follicles [34, 35]. The continuous proliferation and E2 secretion of GCs promotes the growth of follicles [36, 37], while the excessive apoptosis of GCs causes follicular atresia [38], retards the development of follicles, and even leads to the delay of sexual maturity [39] and reduction of reproductive performance in mammals [40]. The transcription factor *E2F1*, a member of E2F family, has been highly suggested to be involved in the ovarian follicle development by regulating the proliferation and E2 secretion of GCs in cattle [41]. Previous studies have recommended that the *RSPO2* gene regulates the GC proliferation and promotes follicular development in humans [32] and mice [33]. Altering the DNA methylation status of GCs is involved in ovarian follicle development by regulating the expression level of genes, e.g., *AKR1C3*, *HAPLN1*, and *PTGER1* [27]. In this study, we found that the hypomethylation of −758/−749 and −563/−553 regions in *RSPO2* promoter facilitated the binding of *E2F1* and promoted the transcription of *RSPO2*, which further promoted the proliferation and steroid hormone secretion of GCs, inhibited the apoptosis of GCs, and ultimately promoted follicular development and pubertal initiation (Fig. 8).

DNA methylation is a widely studied epigenetic phenomenon, usually associated with gene silencing [42]. 5-Aza-CdR reduced the hypermethylation level of *RSPO2* and induced its re-expression in gastric cancer cell lines [43]. In this study, we found that the expression level of *RSPO2* was negatively correlated with the methylation level of the promoter CGI during follicular development. (Fig. 1B, C). Moreover, both inhibition of DNA methylation (Fig. 1E) and *DNMT1* knockdown (Fig. 7C) reduced the methylation levels of *RSPO2* promoter and promoted the mRNA and protein expression levels of *RSPO2* in porcine GCs (Figs. 1D and 7B). Thus, we hypothesized that DNA methylation might regulate *RSPO2* expression level in porcine GCs. To further explore the core regions of DNA methylation regulating *RSPO2* expression level, the *RSPO2* promoter was segmented (Fig. 1A). The dual-luciferase activity results showed that the DNA hypermethylation of the CGI2 region induced a prominent lowering of *RSPO2* promoter activity (Fig. 1F). These observations indicated that DNA methylation status of CGI2 region might regulate *RSPO2* expression level in porcine GCs.

Moreover, we explored how DNA methylation regulated the expression level of *RSPO2*. Numerous studies have shown that the hypermethylated state of CG dinucleotide may regulate gene expression by hindering the binding of transcription factors [44–46]. In temozolomide-sensitive glioblastoma cell, the CGI hypermethylation of SNHG12 promoter blocked the occupancy of SP1 and inhibited the expression level of SNHG12 [44]. Thus, we predicted the potential transcription factor-binding site on all CG dinucleotide within the CGI region of *RSPO2* promoter and found that transcription factor *E2F1* had the most binding sites in this region (Fig. 7A). Moreover, *DNMT1* knockdown reduced the methylation level of *E2F1*-binding sites at CGI2 (Fig. 7C) and significantly increased the expression of *RSPO2* (Fig. 7B). That *DNMT1* knockdown promoted the binding of *E2F1* to the *RSPO2* promoter CGI2 region was further determined by ChIP (Fig. 7D) and ChIP-qPCR (Fig. 7E). Further, *E2F1* overexpression significantly promoted the activity of unmethylated CGI2 region in *RSPO2* promoter compared to methylated CGI2 region in porcine GCs (Fig. 7G). These observations indicated that DNA methylation might partially inhibit the expression level of *RSPO2* by hindering *E2F1* binding to the CGI2 region of *RSPO2* promoter.

It was widely known that abnormal proliferation and apoptosis can affect E2 secretion of GCs and follicular development. Our data showed that *RSPO2* promoted the proliferation of GCs, inhibited the apoptosis of GCs, and promoted the secretion of E2. *PCNA* [47], *CDK1* [48], and *CCND1* [49], all known to be proliferation regulators, have been confirmed to promote GC proliferation and follicular development in mammals. The aromatase encoded by *CYP19A1* is a key rate-limiting enzyme for E2 biosynthesis [50, 51]. Upregulation of *CYP19A1* expression stimulates E2 release and inhibits porcine GC apoptosis and follicular atresia [52]. Activation of *BAX* stimulates *Caspase3/9* and then promotes the cleavage of *PARP*, thereby inducing the apoptotic process [53]. The decreased expression of *BAX* inhibited GC apoptosis in rat [54], and knockout of *BAX* in mice resulted in an increase in ovarian follicle numbers [55]. Decreased expression of *Caspase3* inhibited apoptosis of premature ovarian insufficiency rat ovaries to restore ovarian function and structure [56]. Further, we detected the expression level of several key genes in the signaling pathways associated with proliferation, apoptosis, and steroid hormone secretion after overexpression or knockdown of *RSPO2* in GCs. Among these genes, the expression levels of proliferation markers *PCNA*, *CDK1*, and *CCND1* were significantly increased by pcDNA3.1-*RSPO2* (Fig. 2C, D), and these were significantly decreased by *RSPO2*-siRNA (Fig. 2G, H). The expression levels of pro-apoptotic genes *Caspase3*, cleaved *Caspase3*, cleaved *Caspase9*, cleaved *PARP*, and *BAX* were significantly decreased by pcDNA3.1-*RSPO2* (Fig. 3B, C), and these were significantly increased by *RSPO2*-siRNA (Fig. 3E, G). The mRNA and protein levels of steroid hormone secretion markers *CYP19A1* and *HSD17B1* were significantly increased by pcDNA3.1-*RSPO2*, and these were significantly decreased by *RSPO2*-siRNA (Fig. 4A, B). Overall, *RSPO2* might promote the proliferation and inhibit the apoptosis and secretion of E2 in porcine GCs.

Previous studies have confirmed that healthy follicles were characterized with a well-vascularized follicular wall and the clarity of follicular fluid, and atretic follicles were characterized with no blood vessels and the opacification of follicular fluid [57, 58]. The apoptosis of GCs is the main cause of follicular atresia and selection [59, 60]. In this study, we found that *RSPO2* knockdown promoted apoptosis of porcine follicular GCs (Fig. 5D), the loss of follicular blood vessels, and the opacification of follicular fluid (Fig. 5C). Similarly, *RSPO2* knockdown reduced the E2 concentration in serum (Fig. 6D), blocked most of the follicles in the antral follicle stage (Fig. 6E), and promoted the apoptosis of follicular GCs in mice (Fig. 6F). Additionally, studies have indicated that *RSPO2*-LGRs could activate and enhance the Wnt/β-catenin signaling pathway [61] to participate in GC apoptosis [62], proliferation, and follicle development [33]. In this study, we found that inhibition of *RSPO2* decreased the mRNA and protein expression levels of *LGR4* and *CTNNB1* in the porcine follicles (Fig. 5A, B) and mice ovaries (Fig. 6A, B). Interestingly, we also found that *RSPO2* knockdown delayed the onset of mouse puberty (Fig. 6C). Therefore, we speculate that inhibition of *RSPO2* may delay the initiation of puberty by inhibiting follicle development. In summary, inhibition of *RSPO2* might promote the apoptosis of follicular GCs through the Wnt signaling pathway, thereby inhibiting the development of follicles in mammals.

## Materials and methods

### Cell and tissue culture

The ovaries of pre-puberty sows were collected from a local slaughterhouse and transported to the laboratory using phosphate-buffered saline (PBS) containing penicillin (100 IU/mL) and streptomycin (100 μg/mL) (Invitrogen, Shanghai, China). Subsequently, the GCs were aspirated by inserting a syringe into a 3–5-mm follicle and washed twice with PBS. The cells were then seeded into culture flasks containing 10% fetal bovine serum (Hyclone, Logan, UT, USA) in Dulbecco’s modified Eagle’s medium (DMEM; Hyclone, Logan, UT, USA) and 100 IU/mL penicillin and 100 μg/mL streptomycin and finally incubated at 37 °C under 5% CO_2_.

Antral follicles 3–5 mm in diameter were removed from the ovaries using forceps and scalpels. Then the follicles were cleaned with PBS and follicular culture medium, respectively. Follicular culture medium was serum-free DMEM/F12. Twenty-four-well plates were used for culture, with one follicle per well, and incubated at 38.5 °C under 5% CO_2_.

### Quantitative reverse transcription PCR

TRIzol reagent (TaKaRa, Tokyo, Japan) was used to extract total RNA from the sample, and then the RevertAid First Strand cDNA Synthesis Kit (Thermo Scientific, Waltham, MA, USA) was used to reverse-transcribe the mRNAs. Maxima SYBR Green qRT-PCR Master Mix (2×) (Thermo Scientific, Waltham, MA, USA) was used to quantify the relative expression levels of mRNAs in the CFX96 Touch Real-Time PCR system (Bio-Rad, Berkeley, CA, USA). Using the expression level of glyceraldehyde phosphate dehydrogenase as endogenous control, the relative expression level of genes was calculated with the 2^−ΔΔct^ method. The primer sequences used in pig are listed in Table 1 and that used in mouse are listed in Table 2.

**Table 1:** Primers used for qRT-PCR in pig.

Gene name	Primer sequences (5’ → 3’)	Size (bp)	Accession number
RSPO2	F: GATGGAGACGCAGTAAGCGA R: CATATCTGGGGCTCGGTGTC	197	NM_001293141.1
Caspase3	F: ACATGGAAGCAAATCAATGGAC R: TGCAGCATCCACATCTGTACC	154	NM_214131.1
Caspase8	F: GAGCCTGGACTACATCCCAC R: GTCCTTCAATTCCGACCTGG	283	NM_001031779.2
Caspase9	F: GCTGAACCGTGAGCTTTTCA R: CCTGGCCTGTGTCCTCTAAG	161	XM_003127618.4
BAX	F: ACTTCCTTCGAGATCGGCTG R: AAAGACACAGTCCAAGGCGG	184	XM_013998624.2
BCL2	F: GATGCCTTTGTGGAGCTGTATG R: CCCGTGGACTTCACTTATGG	145	XM_021099593.1
PCNA	F: TCGTTGTGATTCCACCACCAT R:TGTCTTCATTGCCAGCACATT	278	NM_001291925.1
CDK1	F: AGGTCAAGTGGTAGCCATGAA R: CATGAACTGACCAGGAGG	225	NM_001159304.2
CDK2	F: AAAGATCGGAGAGGGCACG R: GCAGTACTGGGTACACCCTC	121	NM_001285465.1
CDK4	F: CCTCCCGGTATGAACCAGTG R: TGCTCAAACACCAGGGTCAC	277	NM_001123097.1
CCNA1	F: GCGCCAAGGCTGGAATCTAT R: CCTCAGTCTCCACAGGCTAC	196	XM_005668339.3
CCNA2	F: GTACTGAAGGCCGGGAACTC R: AGCTGGCCTCTTTTGAGTCT	192	NM_001177926.1
CCNB1	F: ACGGCTGTTAGCTAGTGGTG R: GAGCAGTTCTTGGCCTCAGT	236	NM_001170768.1
CCNB2	F: TGGAAATCGAGTTACAACCAGA R: TGGAGCCAACATTTCCATCTGT	151	NM_001114282.1
CCND1	F: CTTCCATGCGGAAGATCGTG R: GGAGTTGTCGGTGTAGATGC	234	XM_021082686.1
CCND2	F: TTCCCCAGTGCTCCTACTTC R: CACAACTTCTCAGCCGTCAG	259	NM_214088.1
CCNE1	F: AGCCTGTGAAAACCCCTGTT R: TCCAGAAGAATCGCTCGCAT	252	XM_005653265.2
CCNE2	F: GGGGGATCAGTCCTTGCATT R: AGCCAAACATCCTGTGAGCA	154	NM_001243931.1
CYP19A1	F: CTGAAGTTGTGCCTTTTGCCA R: CTGAGGTAGGAAATTAGGGGC	139	NM_214429.1
STAR	F: CGACGTTTAAGCTGTGTGCT R: ATCCATGACCCTGAGGTTGGA	136	NM_213755.2
HSD17B1	F: GTCTGGCATCTGACCCATCTC R: CGGGCATCCGCTATTGAATC	166	NM_001128472.1
HSD3B1	F: ATCTGCAGGAGATCCGGGTA R: CCTTCATGACGGTCTCTCGC	216	NM_001004049.2
E2F1	F: GACTCCTCGCAGATCGTCAT R: CGCCTCCAGGCCAAACATAG	263	XM_021077692.1
LGR4	F: GACCGTCGGGTAGATTGCTC R: GCTCCTCTAGAAAAGGGAAGTT	145	XM_021083233.1
CTNNB1	F: AAGCGGCTGTTAGTCACTGG R: TGAGCGCGAGTCATTGCATA	220	NM_214367.1
GAPDH	F: TCACCAGGGCTGCTTTTAACT R: CTTGACTGTGCCGTGGAACT	131	NM_001206359.1

**Table 2:** Primers used for qRT-PCR in mouse.

Gene name	Primer sequences (5’ → 3’)	Size (bp)	Accession number
RSPO2	F: CTCGCACAACGCCCTTTCT R: GTCGAGGAAATGACGGGCT	271	NM_001357957.1
Caspase3	F: TGGCGTGTGCGAGATGAG R: TTGTTGTTCTCCATGGTCAC	211	NM_009810.3
BAX	F: GCACGTCCACGATCAGTCAC R: CACTCGCTCAGCTTCTTGGT	261	NM_007527.3
BCL2	F: TTCAGCATTGCGGAGGAAGT R: GCACTTCAAGTCCCGACTCC	267	NM_009741.5
PCNA	F: GTGAACCTGCAGAGCATGGA R: TGGTGCTTCGAATACTAGTGC	216	NM_011045.2
CDK1	F: TGGGGTGTTGTTTCCACAGTT R: AGGGGCTGAGACCAATGGAG	268	NM_007659.4
CCND1	F: GCCATCCATGCGGAAAATCG R: GGCAGTCAAGGGAATGGTCT	205	NM_001379248.1
CYP19A1	F: CCACCACTGCTTTCTTCCCAT R: CACTTCCAATCCCCATCCACA	283	NM_007810.4
HSD17B1	F: AGATTGCCAGCAGACACAACA R: CAACAATGGTCCCTGTGCCTT	273	XM_006532297.3
LGR4	F: CCATTCGTGGACTGAGTGCT R: GTTGGTGAATGCGAAGTCGG	227	NM_172671.2
CTNNB1	F: TCCTTCACGCAAGAGCAAGT R: ATTGCACGTGTGGCAAGTTC	242	NM_007614.3

### EdU and MTT assay

The Cell-Light^TM^ Edu Apollo 567 In Vitro Kit (RiboBio, Guangdong, China) was used to analyze cell proliferation. Briefly, GCs were cultured in 48-well plates and transfected with plasmid for 24 h. GCs were incubated at room temperature with 50 μM EDU for 2 h, washed twice with PBS, and then incubated with 80% acetone for 30 min. After GCs were washed twice by PBS, 0.5% Triton X-100 was added for 10 min, 1× Apollo was incubated in darkness for 30 min, and Hoechst was incubated for 30 min. Finally, three fields were randomly selected from each well and GCs were counted under an inverted fluorescence microscope.

The MTT Cell Proliferation and Cytotoxicity Assay Kit (Beyotime, Shanghai, China) was used to analyze cell proliferation. Briefly, GCs were cultured in 10 μL (5 mg/mL) MTT solution for 4 h and then added 100 μL Formazan solution for 4 h in 96-well plates. Finally, the absorbance of 570 nm was measured.

### Flow cytometry

The Annexin V-FITC Apoptosis Detection Kit (BioVision, Milpitas, CA, USA) was used to analyze cell apoptosis. Briefly, GCs were cultured in 6-well plates and transfected with plasmids for 24 h. The collected cells were centrifuged at 1000 rpm for 5 min, the supernatant was discarded, and washed twice with PBS. Then, 500 μL of 1× Annexin V buffer was added to gently resuspend the cells, and 5 μL of Annexin V–fluorescein isothiocyanate and 5 μL of propidium iodide (PI) staining solution were added and mixed. Finally, flow cytometry was performed after incubation for 15 min at room temperature in darkness. For results, the figure has four quadrants, the lower right quadrant is annexin-positive/PI-negative early apoptotic cells, the upper right quadrant is annexin-positive/PI-positive late apoptotic cells, the lower left quadrant is living cell, and the upper left quadrant is mechanical injury cells. In this study, the apoptotic ratio of GCs is the sum of early and late apoptosis.

### Enzyme-linked immunosorbent assay (ELISA)

The concentration of E2 in the serum of mice and supernatant of cell culture medium was detected using a mouse or porcine E2 ELISA Kit (Jingmei Biotechnology, Jiangshu, China) performed according to the manufacturer’s instructions. Fifty microliters of standard with different concentrations were added into the standard well, and 50 μL of samples to be tested were added into the sample well. Then 100 μL of horseradish peroxidase (HRP) was added to each well and incubated for 60 min at 37 °C. After cleaning, 50 μL of substrate A and B were added and incubated for 15 min at 37 °C. Finally, termination solution was added to measure the optical density value at 450 nm.

### Plasmid construction and dual-luciferase reporter assay

The promoter (−2232/+66 bp), non-CpG island 1 (NCGI1) (−1540/−1357 bp), CGI1 (−1345/−1165 bp), NCGI2 (−1187/−1003 bp), CGI2-1 (−1014/−721 bp), CGI2–2 (−709/−362 bp), and NCGI3 (−327/−76 bp) regions of *RSPO2* were amplified from genomic DNA of GCs, followed by cloning into the pGL3 vector to obtain pGL3-*RSPO2*, pGL3-NCGI1, pGL3-CGI1, pGL3-NCGI2, pGL3-CGI2-1, pGL3-CGI2–2, and pGL3-NCGI3 recombinant vectors. The pGL3-basic and pGL3-control were used as the control group. Then the CpG transmethylase M.SssI (M0226, NEW ENGLAND Biolabs, Beijing, China) was incubated with these 9 vectors for 1 h at 37 °C to methylate all CpG residues and the corresponding unmethylated group was incubated with nuclease-free water. The BstU I endonuclease was incubated with these vectors for 1 h at 60 °C to detect the methylation status.

The CDS region of *RSPO2* and *E2F1* gene were amplified from cDNA of GCs and cloned into the expression vector pcDNA3.1 (Invitrogen). These vectors were purified by the HiPure Plasmid EF Micro Kit (P1111-03, Magen, Guangzhou, China). All siRNAs were designed and synthesized by RiboBio (Guangzhou, China). Plasmids were transfected into 80% confluent GCs for 24 h using Lipofectamine^TM^ 3000 Reagent (Thermo Scientific, Waltham, MA, USA). All luciferase activities were measured with the BioTek Synergy 2 multifunctional microplate reader (BioTek, Winooski, VT, USA) by using a Dual-luciferase Reporter Assay Kit (Promega, Madison, WI, USA) and normalized to Renilla luciferase activity. The primer sequences are listed in Table 3.

**Table 3:** Primers used for vector construction.

Primer name	Primer sequences (5’ → 3’)	Size (bp)
pcDNA3.1-RSPO2	F: CCAAGCTTTCCTTTGCCCTCATCATCCTG R: GGGGTACCAGCTAGGAAGACGCTGTGTTG	693
pcDNA3.1-E2F1	F: CCAAGCTTCCCCATCCTGCGATTGGC R: GGGGTACCCCCATCAGAAATCCAGGGGG	1511
pGL3-RSPO2	F: CGACGCGTACCTCAGGGGCTGAATTTTCTT R: CCCTCGAGTTGGCAGTGGCTGTAATCCAT	2299
pGL3-NCGI1	F: CGACGCGTGGATAAGCAAAGGATTAGAATA R: CCCTCGAGCCAGGCTCTGACTGCTGCCTCT	183
pGL3-CGI1	F: CGACGCGTAAGGTCTAGGAGAAACCATCAG R: CCCTCGAGTGAGGAGTAGCTGAGGTGG	180
pGL3-NCGI2	F: CGACGCGTACCTCAGCTACTCCTCAATTTA R: CCCTCGAGGTCCAAGGCAACTAAGTCAG	184
pGL3-CGI2-1	F: CGACGCGTTTGGACCACAGCACCACCTAC R: CCCTCGAGCAGCGCCTAGCTGGAGCGCAGT	293
pGL3-CGI2-2	F: CGACGCGTCTCCGCGCACTTCGAAACCACT R: CCCTCGAGGACCCATAGGCTCCGACCCGG	347
pGL3-NCGI3	F: CGACGCGTCTCCAAGTTGCCGGCAGCTG R: CCCTCGAGCTGCGGTGCAAGACTCAGAGG	251

Sequences underlined represent the enzyme-cutting sites.

### Animals and lentivirus delivery

Four-week-old female C57BL/6J mice (*n* = 15) were purchased from Guangdong Medical Laboratory Animal Center (Guangzhou, China) and were randomly divided into blank (*n* = 5), sh-NC (*n* = 5), and sh-*RSPO2* (*n* = 5) groups with 5 in each group.

The CDS region or siRNA of *RSPO2* was inserted into a GV208 lentivirus vector containing a CMV-driven EGFP reporter gene and a Ubi promoter upstream of restriction sites (Age I and BamH I). All constructs were verified by sequencing. Then recombinant lentiviruses were produced by co-transfecting 293T cells. GFP expression was measured in 293T cells to determine the virus titers, expressed as transducing units (TU) per mL. The titers of lentiviral vector that overexpress or interfere with *RSPO2* (LV-*RSPO2* or sh-*RSPO2*) was approximately 1E + 9 TU/ml. In all, 1 × 10^7^ TU of lentivirus was then injected into the mice of each group (*n* = 5) through intraperitoneal injection. The injection was given once a week for 3 weeks.

### Bisulfite sequencing PCR

Genomic DNA was extracted from GCs and follicles with a Tissue DNA Kit (D3396-02, Omega Bio-tek, USA). The purified DNA was exposed to bisulfite with an EZ DNA Methylation-Gold^TM^ Kit (D5006, ZYMO RESEARCH, CA, USA) according to the manufacturer’s protocol. Then the corresponding fragment was amplified by BSP primer using the bisulfite-converted DNA as template. Finally, the sequencing results are compared with the original sequence through the QUMA website (http://quma.cdb.riken.jp/) and plotted. Ten clones were needed for each sample to calculate the methylation rate. The specific primers used for BSP are listed in Table 4.

**Table 4:** Primers used for BSP.

Primer name	Primer sequences (5’ → 3’)
NCGI1	F1: GGATAAGCAAAGGATTAGAATACTCT R1: CCAGGCTCTGACTGCTGCCTCT
CGI1	F2: AAGGTCTAGGAGAAACCATCAGTCGG R2: TAAATTGAGGAGTAGCTGAGGTGG
NCGI2	F3: ACCTCAGCTACTCCTCAATTTAGC R3: CTGTGGTCCAAGGCAACTAAGTCAG
CGI2-1	F4: TTGGACCACAGCACCACCTACTTAT R4: TAACAGCGCCTAGCTGGAGCGCAGT
CGI2-2	F5: CTCCGCGCACTTCGAAACCACTGCA R5: TGCGGACCCATAGGCTCCGACCCGG
NCGI3	F6: CTCCAAGTTGCCGGCAGCTGTGAGT R6: TGCGCTGCGGTGCAAGACTCAGAGG

### TUNEL assay

Apoptotic cells were detected by the TUNEL assays using a One Step TUNEL Apoptosis Assay Kit (Beyotime Biotech, Jiangshu, China) according to the manufacturer’s protocol. Briefly, the different treated follicles were made into paraffin sections and then treated with xylene, ethanol, and protease K, respectively. After being washed by PBS, the sections were treated with the freshly prepared TUNEL reaction mixture. Images were obtained by using a Nikon ECLIPSE Ti2 fluorescence microscope.

### ChIP assay

ChIP assay was conducted using the Pierce^TM^ ChIP Kit (ThermoFisher, Rockford, IL, USA) according to the manufacturer’s instructions. The cultured GCs were incubated with 1% formaldehyde at room temperature for 10 min. After GCs were quenched with glycine, the chromatin fragments of GCs were extracted with automatic ultrasonic crushing apparatus. The specific E2F1 antibody (ab179445, Abcam, Cambridge, MA, USA) and IgG antibody (12–370, Millipore) were then incubated with the cell fragmentation solution at 4 °C overnight. The immunoprecipitated DNA was purified for PCR and qPCR analyses, and the specific primers are listed in Table 5.

**Table 5:** Primers used for ChIP-PCR.

Primer name	Primer sequences (5’ → 3’)
RSPO2 site 1	F: CTGCGCTGTGGTTGGAACC R: AGGCGCCTCCTGACTGTGC
RSPO2 site 2	F: ACAACTGGACAGTTGGTCAC R: GCGTCCCGCTGCGGGTGC
RSPO2 site 3	F: CCTGGCGCGACGACTTAG R: ATGCGCAGCCCGATTTGG

### WB analysis

The total protein was extracted and the concentration was detected by a BCA Protein Assay Kit (Vigorous Bio-technology Beijing Co., Ltd., Beijing, China). Equal amounts of sample protein were electrophoresed on 4–20% sodium dodecyl sulfate-polyacrylamide gel electrophoresis gels and transferred to polyvinylidene difluoride (PVDF) membranes. Then the PVDF membranes were sealed with Tris-buffered saline-Tween (TBST) solution, which contained 5% skimmed milk at room temperature for 1–2 h. After the PVDF membranes were washed with TBST for 3 times, the membranes were incubated overnight at 4 °C with the following diluted primary antibody: RSPO2 (17781-1-AP, Proteintech, 1:1000), E2F1 (ab179445, Abcam, 1:2000), PCNA (10205-2-AP, Proteintech, 1:2000), CDK1 (19532-1-AP, Proteintech, 1:1000), CCND1 (26939-1-AP, Proteintech, 1:1000), Caspase3 (19677-1-AP, Proteintech, 1:1000), cleaved Caspase3 (#9661, CST, 1:1000), cleaved Caspase8 (#8592, CST, 1:1000), cleaved Caspase9 (#9509, CST, 1:1000), cleaved PARP (#5625, CST, 1:1000), BAX (50599-2-Ig, Proteintech, 1:5000), BCL2 (12789-1-AP, Proteintech, 1:2000), CYP19A1 (bs-0114R, Bioss, 1:1000), HSD17B1 (bs-6603R, Bioss, 1:1000), LGR4 (20150-1-AP, Proteintech, 1:1000), CTNNB1 (51067-2-AP, Proteintech, 1:10,000), and Tubulin (11224-1-AP, Proteintech, 1:5000). The membranes were washed with TBST for 3 times and incubated with goat anti-rabbit IgG H&L (HRP) (ab205718, Abcam, 1:10,000) or goat anti-mouse IgG H&L (HRP) (ab6789, Abcam, 1:5000) for 2 h at room temperature. The blots were visualized by the Odyssey Fc Imaging System (LI-COR Biosciences, Lincoln, NE), and the intensity of protein bands was measured by the NIH ImageJ software.

### Statistical analysis

In all the panels, data are shown as mean ± standard deviation from at least three biological replicates. Statistical significance of differences between means was analyzed by Student’s *t* test (**P < 0.05 and *P < 0.01).
